# Editorial: Emerging fast medical imaging techniques in radiology

**DOI:** 10.3389/fradi.2026.1948195

**Published:** 2026-08-05

**Authors:** Zhifeng Chen, Zhicheng Peng, Mingfeng Jiang, Jianfeng Zhou, Lijun Lu, Xiaoyun Liang

**Affiliations:** 1 Institute of Research and Clinical Innovations, Neusoft Medical Systems Co., Ltd., Hangzhou, Zhejiang, China; 2 Department of Chemistry and Biochemistry, Texas Tech University, Lubbock, TX, United States; 3 School of Computer Science and Technology, Zhejiang Sci-Tech University, Hangzhou, Zhejiang, China; 4 Department of Technology and Equipment, Taicang Wugang Dock Co., Ltd., Taicang, Jiangsu, China; 5 School of Biomedical Engineering, Southern Medical University, Guangzhou, Guangdong, China; 6 Institute of Research and Clinical Innovations, Neusoft Medical Systems Co., Ltd., Shanghai, China

**Keywords:** artificial intelligence, computed tomography, deep learning, fast imaging, magnetic resonance imaging, quantitative imaging, radiomics, spectral CT

The field of medical imaging is undergoing a rapid transformation driven by the dual imperatives of reducing acquisition times and enhancing diagnostic precision (1–3). As clinical workflows demand ever-greater throughput without compromising image quality, emerging fast imaging techniques (4–11), spanning hardware innovation (12, 13), algorithmic reconstruction (11, 14, 15), and artificial intelligence (AI) (2, 3, 10, 16), have become central to modern radiology. This Research Topic, “Emerging Fast Medical Imaging Techniques in Radiology,” brings together 15 articles across several Frontiers journals. Collectively, these contributions underscore the rapid evolution and expanding scope of cutting-edge imaging methodologies in clinical practice. The contributions span computed tomography (CT), magnetic resonance imaging (MRI), ultrasound, nuclear medicine, and hybrid imaging, covering applications from oncologic staging and cardiovascular assessment to neurodegenerative biomarker discovery and infectious disease surveillance across diverse clinical settings from tertiary academic centers to resource-limited environments. Together, they offer a multifaceted view of how speed and quantitative precision can be achieved simultaneously across the radiological spectrum.

## Purpose and scope of this research topic

This Research Topic aims to provide a comprehensive and integrative perspective on fast imaging innovations across the radiological spectrum. Specifically, it seeks to showcase advances in AI-driven acquisition and reconstruction, quantitative radiomics, spectral CT, advanced MRI, and hybrid nuclear medicine. Key objectives include demonstrating how these technologies reduce scan times and radiation or contrast doses while preserving or enhancing diagnostic accuracy, and illustrating their clinical applications ranging from oncologic staging to acute stroke triage. The Research Topic encompasses original research, systematic reviews, meta-analyses, and clinical case reports, providing a multidimensional perspective on the future of fast imaging.

## AI and deep learning for accelerated acquisition and interpretation

A central theme across this Research Topic is the application of AI and deep learning to accelerate both image acquisition and interpretation. In cardiac CT, Zhu et al. combined the Snapshot Freeze motion correction algorithm with single-heartbeat acquisition to achieve diagnostic-quality coronary CT angiography in patients with atrial fibrillation, a population in which conventional multi-beat protocols frequently suffer from stair-step artifacts and non-diagnostic segments. For acute stroke triage, Tsuji et al. validated an automated deep-learning model for hyperdense artery sign detection on non-contrast CT across multiple centers, demonstrating high sensitivity and specificity as a pre-CTA alert system that can expedite large vessel occlusion identification and parallelize interventional workflow. In chest radiography, Kai et al. developed a vision Transformer-based system to classify cardiopulmonary abnormalities in resource-limited settings, offering rapid AI-driven screening where radiologist availability is constrained. In ultrasound, Chen et al. introduced a two-stage deep learning method that automated both segmentation and classification of lumbar transverse views, reducing inter-operator variability and facilitating faster, safer spinal anesthesia guidance. Additionally, Wang et al. validated deep learning-based reconstruction for 5.0 T knee MRI, where CNN-based denoising substantially improved signal-to-noise ratio, image sharpness, and diagnostic confidence compared with conventional parallel imaging, without any prolongation of acquisition time. Collectively, these studies demonstrate that AI integration spans the entire imaging chain, from acquisition optimization to post-processing, and is applicable across diverse modalities and clinical scenarios.

## Radiomics and quantitative biomarkers

Radiomics and quantitative texture analysis represent another prominent axis of innovation. Renton et al. leveraged multiparametric MRI radiomics to predict disease-free survival and high-risk histopathological features in endometrial cancer, establishing a non-invasive prognostic framework for personalized risk stratification. A meta-analysis by Yang et al. synthesized the growing body of evidence on MRI-based radiomics for predicting lymphovascular space invasion in cervical cancer, providing pooled sensitivity and specificity estimates that support clinical translation. In adrenal imaging, Tarbaeva et al. demonstrated that CT texture analysis can non-invasively predict mitotic activity and morphological variants of adrenocortical carcinoma, offering a preoperative tool to distinguish aggressive from indolent tumors. Complementing these findings, Sun et al. constructed a prognostic survival nomogram for colorectal cancer by integrating CT texture features with clinicopathological variables, achieving superior predictive performance over clinical models alone. Together, these studies highlight how standardized radiomic pipelines could convert routine images into quantitative biomarkers that extend diagnostic utility without imposing additional acquisition burden on patients.

## CT protocol optimization and hybrid imaging

Advances in CT technology and protocol optimization feature prominently in this collection. Li et al. achieved a 20% reduction in iodinated contrast dose and a 4% reduction in radiation dose by employing a low-threshold trigger with saline chaser in CT angiography, a clinically significant step toward safer imaging, particularly for patients with renal impairment. Zhuang et al. demonstrated the diagnostic value of dual-layer spectral detector CT in characterizing primary adrenal lymphoma, a rare and diagnostically challenging entity, by leveraging spectral parameters including virtual monoenergetic imaging, iodine density maps, and effective atomic number to differentiate it from mimics. In nuclear medicine, Dhingra et al. documented 99mTc-MDP bone scintigraphy with SPECT/CT in post-COVID mucormycosis, illustrating the continued importance of hybrid functional imaging in detecting invasive fungal complications in an era of emerging infectious threats. These studies underscore that protocol refinement and modality integration remain powerful levers for improving diagnostic safety and accuracy.

## Emerging computational and quantitative frontiers

This collection further extends to cutting-edge computational frontiers. Glenn et al. evaluated quantum computing-based auto-contouring algorithms for radiation therapy treatment planning on abdominal MRI, representing one of the earliest explorations of quantum image processing in oncology. Although still in the prototype stage, this work signals a future paradigm in which quantum-accelerated segmentation could overcome the computational bottlenecks that currently delay treatment planning. In quantitative MRI, Wang et al. employed 3.0 T quantitative susceptibility mapping (QSM) to investigate age-dependent iron deposition patterns in deep gray matter nuclei, thus establishing normative susceptibility baselines with direct implications for detecting early neurodegenerative changes. Zhou et al. introduced time-intensity curve parametric imaging as a novel quantitative biomarker for contrast-enhanced ultrasound in prostate cancer, significantly improving diagnostic accuracy and reducing inter-rater variability across experience levels. These contributions have collectively illustrated how novel computational approaches can extract previously inaccessible information from routine clinical data.

## Cross-cutting themes and concluding perspective

Several cross-cutting themes emerge from this collection. First, AI integration spans the entire imaging chain, from acquisition protocol optimization and motion correction to reconstruction, segmentation, and clinical decision support. Second, quantitative biomarkers derived from radiomics, spectral CT, QSM, and parametric mapping have been increasingly bridging the gap between qualitative visual assessment and personalized, data-driven medicine. Third, dose reduction, whether in contrast agents, ionizing radiation, or scan duration, is achievable without diagnostic compromise, underscoring that “fast” and “safe” are complementary rather than competing objectives. Finally, the geographic and institutional diversity of the contributing authors reflects a global research community united by the shared mission of making medical imaging faster, safer, thus conferring clinical benefits on patients globally. Collectively, the 15 manuscripts in this Research Topic underscore the multifactorial nature of innovation in fast medical imaging, encompassing hardware engineering, computational algorithms, and clinical insight. These studies demonstrate that meaningful progress in radiology arises not from any single technological breakthrough but from the convergence of AI-driven reconstruction, quantitative biomarker extraction, protocol optimization, and multi-modal integration. Future research should prioritize large-scale, multi-center validation, longitudinal outcome studies, and the integration of these technologies into routine clinical workflows. Strengthening interdisciplinary collaboration between computer scientists, medical physicists, and clinicians will be essential to fully realize the clinical potential of these emerging technologies. It is our hope that this collection will catalyze further innovation at the intersection of speed, safety, and diagnostic precision in radiology.
